# Assessment of concurrent neoplasms and a paraneoplastic association in MOGAD

**DOI:** 10.1002/acn3.52301

**Published:** 2025-02-11

**Authors:** Young Nam Kwon, Nanthaya Tisavipat, Yong Guo, Stephanie B. Syc‐Mazurek, Ji Yeon Han, Jun‐Soon Kim, Kyomin Choi, Seong‐il Oh, Seok‐Jin Choi, Eunhee Sohn, Jeeyoung Oh, Seung Woo Kim, Ha Young Shin, Byung Chan Lim, Byoung Joon Kim, Kyung Seok Park, Jung‐Joon Sung, Se Hoon Kim, Sung‐Hye Park, Anastasia Zekeridou, Claudia F. Lucchinetti, Sean J. Pittock, John J. Chen, Eoin P. Flanagan, Sung‐Min Kim

**Affiliations:** ^1^ Department of Neurology Severance Hospital, Yonsei University College of Medicine Seoul Republic of Korea; ^2^ Department of Neurology Mayo Clinic Rochester Minnesota USA; ^3^ Center for MS and Autoimmune Neurology Mayo Clinic Rochester Minnesota USA; ^4^ Department of Pediatrics Inha University Hospital Incheon Republic of Korea; ^5^ Department of Neurology Seoul National University Bundang Hospital, Seoul National University College of Medicine Seongnam Republic of Korea; ^6^ Department of Neurology Soonchunhyang University Cheonan Hospital Cheonan Republic of Korea; ^7^ Department of Neurology Kyung Hee University Hospital, Kyung Hee University College of Medicine Seoul Republic of Korea; ^8^ Department of Neurology Seoul National University Hospital, Seoul National University College of Medicine Seoul Republic of Korea; ^9^ Department of Neurology Chungnam National University College of Medicine, Chungnam National University Hospital Daejeon Republic of Korea; ^10^ Department of Neurology Konkuk University School of Medicine, Konkuk University Medical Center Seoul Republic of Korea; ^11^ Department of Pediatrics Seoul National University College of Medicine, Seoul National University Children's Hospital Seoul Republic of Korea; ^12^ Department of Neurology Samsung Medical Center, Sungkyunkwan University School of Medicine Seoul Republic of Korea; ^13^ Department of Pathology Severance Hospital, Yonsei University College of Medicine Seoul Republic of Korea; ^14^ Department of Pathology Seoul National University Hospital, Seoul National University, College of Medicine Seoul Republic of Korea; ^15^ Department of Laboratory Medicine and Pathology Rochester Minnesota USA; ^16^ Department of Ophthalmology Mayo Clinic Rochester Minnesota USA

## Abstract

Cases of myelin oligodendrocyte glycoprotein (MOG) antibody‐associated disease (MOGAD) co‐occurring with neoplasms have been reported. In this international, retrospective cohort study in South Korea and the USA, 16 of 445 (3.6%) patients with MOGAD had concurrent neoplasm within 2 years of MOGAD onset, resulting in a standardized incidence ratio for neoplasm of 3.10 (95% confidence interval [CI], 1.77–4.81; *P* < 0.001) when compared to the age‐ and country‐adjusted incidence of neoplasm in the general population. However, none of the nine tumor tissues obtained demonstrated MOG immunostaining. The slightly increased frequency without immunohistopathological evidence suggest with true paraneoplastic MOGAD is extremely rare.

## Introduction

Myelin oligodendrocyte glycoprotein (MOG) antibody‐associated disease (MOGAD) has been identified as a distinct autoimmune inflammatory demyelinating disease of the CNS.^1^ Per the 2021 paraneoplastic neurologic syndrome (PNS) diagnostic criteria, MOG‐IgG is designated a low‐risk antibody,^2^ and rare cases of MOGAD with concomitant neoplasm have been reported.^3^, ^4^ We aimed to investigate the frequency of concurrent neoplasm in MOGAD compared to the expected rate in the general population in large MOGAD cohorts from South Korea and the USA, describe the clinical characteristics, determine MOG expression in neoplastic tissues, and apply the criteria for PNS^2^ in patients with MOGAD and tumors.

## Subjects/Materials and Methods

### Study population and data collection

**Figure 1:**
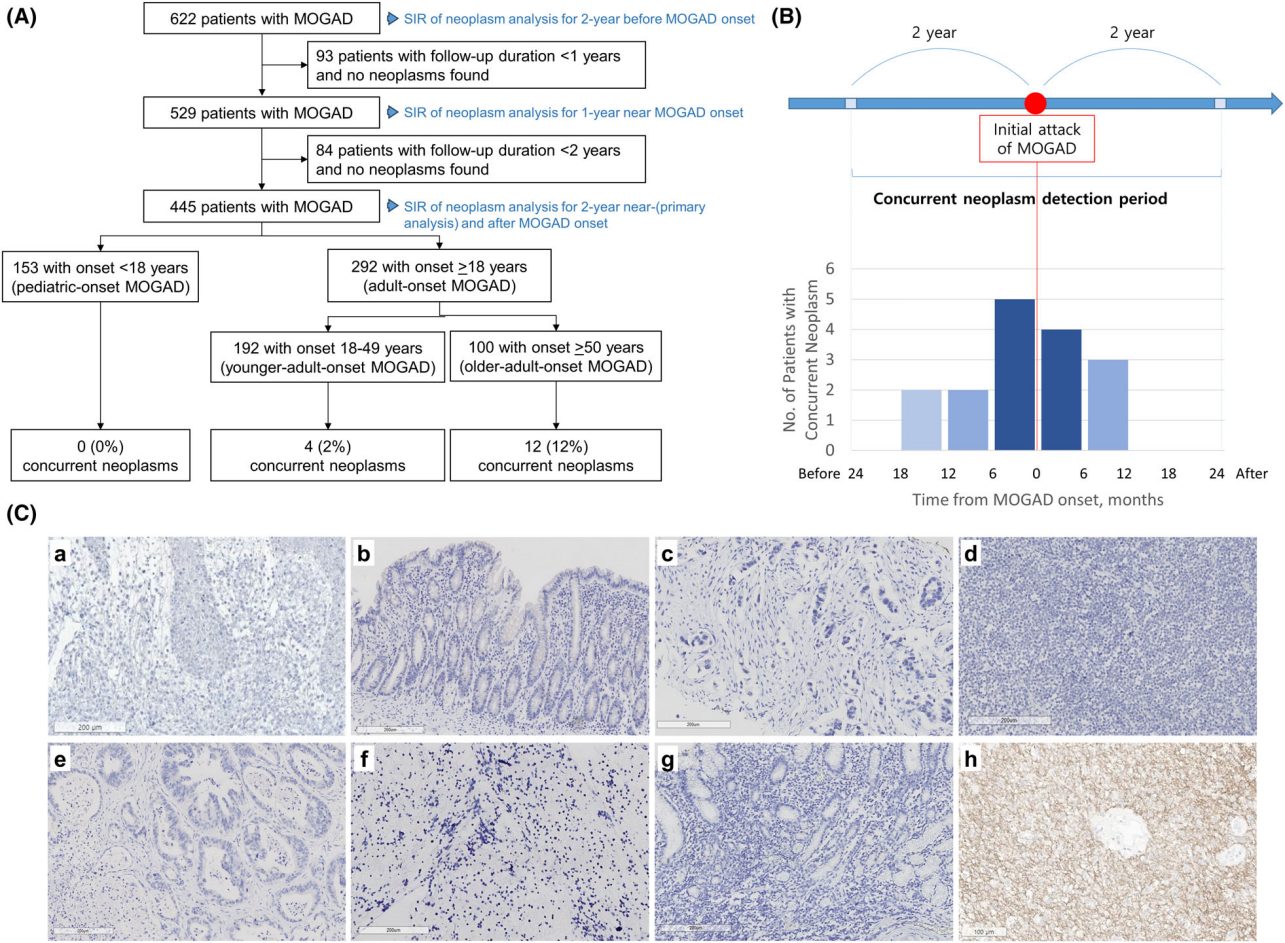
Flowchart of the inclusion process, number of MOGAD patients with concurrent neoplasm, and neoplasm tissue staining for MOG. (A) In our combined cohort of 622 patients with MOGAD, 445 patients with at least 2 years of follow‐up were included in the primary analysis. Concurrent neoplasm was identified in 16 patients (3.6%). In the sensitivity analysis of 529 patients with at least 1 year of follow‐up, concurrent neoplasm within 1 year was found in 14 patients (2.6%). (B) The schematic of concurrent neoplasm definition, and the number of patients with concurrent neoplasm according to the time from MOGAD onset. (C) All staining for MOG in the tissues of concurrent neoplasm were negative: (a) case 2, T‐cell lymphoma; (b) case 4, colon cancer; (c) case 5, pancreatic cancer; (d) case 6, T‐cell lymphoma in small intestine; (e) case 9, colon cancer; (f) case 10, lung cancer; (g) case 16, stomach cancer (Table 2); and (h) glioblastoma as a positive control. MOGAD, myelin oligodendrocyte glycoprotein antibody‐associated disease; No., number.

This is an international, multicenter, retrospective cohort study of 622 MOGAD patients from nine hospitals (Data S1) in South Korea (August 2012–April 2023) and Mayo Clinic, USA (January 2000–April 2023) (Fig. 1A). A total of 605 (97.3%) patients fulfilled the 2023 International MOGAD Panel diagnostic criteria.^1^ MOG‐IgG was tested by live cell‐based immunofluorescence assay as described previously.^1^, ^5^, ^6^, ^7^ The cutoff points for clear positive MOG‐IgG titers were FACS ratio >2.36 using FACSCaliber (BD bioscience)^5^ or >3.65 using Cytomics FC 500 (Beckman Coulter)^1^ in South Korea and titer ≥1:100 or binding index ≥10.0 at the Mayo Clinic Neuroimmunology Laboratory.^1^ According to diagnostic criteria for PNS,^2^ concurrent neoplasm was defined by diagnosis within 2 years of MOGAD onset (Fig. 1B). Screening and investigation for tumors were performed at the physicians' discretion. Patients included for the primary analysis were followed for at least 2 years or diagnosed with concurrent neoplasm within 2 years of MOGAD onset (Fig. 1A). Sensitivity analyses were conducted for the 2‐year pre‐MOGAD period, 2‐year post‐MOGAD, and MOGAD patients with at least 1 year of follow‐up.

Identification of patients with concurrent neoplasm was conducted by chart review together with the Mayo Data Explorer tool at Mayo Clinic.^8^ Data for the South Korea cohort were abstracted from chart review. The following search string “cancer OR carcinoma OR histiocytosis OR leukemia OR lymphoma OR malignancy OR melanoma OR neoplasm OR tumor OR sarcoma OR seminoma OR teratoma OR thymoma” was incorporated into free‐text search in the electronic medical records of MOGAD patients through the Mayo Data Explorer.^8^ After the initial identification of MOGAD patients with neoplasm, chart review was performed for case ascertainment. Squamous cell carcinoma and basal cell carcinoma of skin were excluded due to their localized nature.

### Standardized incidence ratio calculation

Standardized incidence ratio (SIR)^9^ was calculated from the observed number of MOGAD patients with concurrent neoplasm and the expected number extrapolated from age‐ and country‐matched incidence in the general population according to each country's national database (https://kosis.kr/, data from 2019; https://seer.cancer.gov/, data from 2015 to 2019; accessed January 2023). The following formula was used to calculate the SIR of concurrent neoplasm with MOGAD^9^:

$$\mathrm{SIR}=\frac{\text{Observed number of MOGAD patients with concurrent neoplasm}}{\text{Expected number of MOGAD patients with concurrent neoplasm}}$$

### MOG immunostaining in neoplasm tissues and PNS‐care score

Available neoplasm tissues from MOGAD patients were retrieved for MOG immunostaining. Formalin‐fixed paraffin‐embedded (FFPE) 5‐μm thick sections were stained with hematoxylin and eosin (H&E) and immunohistochemistry. The primary antibodies anti‐MOG (Rabbit clone, 1:1000, Abcam, Ab109746) were applied to the sections and incubated overnight at 4°C after steam antigen retrieval with citric acid buffer (pH 6.0, DAKO). The staining was performed with EnVision™ FLEX immunohistochemistry system (DAKO). Negative control was performed by omitting the primary antibody. Normal human spinal cord (in Mayo Clinic) or glioblastoma brain tissue (in South Korea) were used as a positive control for MOG immunohistochemistry. The PNS‐Care score, as defined in the 2021 updated diagnostic criteria,^2^ is applied to patients with concurrent neoplasms.

### Statistical analysis

Baseline characteristics between the South Korea and the Mayo Clinic cohorts were compared by Mann–Whitney *U*‐test for continuous variables and chi‐squared test for categorical variables. SIRs with 95% CIs were calculated according to the Centers for Disease Control and Prevention and the Agency for Toxic Substances and Disease Registry guidelines.^10^ Statistical significance was set at a two‐tailed alpha level <0.05. Statistical analyses were performed using SPSS (version 26 for Windows; IBM, Chicago, IL, USA).

### Standard protocol approvals, registrations, and patient consent

All patients provided consent for the use of their clinical records and pathology specimens for research purposes through the Mayo Clinic Centre for Multiple Sclerosis and Autoimmune Neurology biorepository, Seoul National University Hospital (SNUH), or Severance Hospital. This study was conducted according to the Declaration of Helsinki and was approved by the institutional review board of SNUH (approval number: H‐1907‐163‐1050), Severance Hospital (approval number: 4‐2023‐1060), and Mayo Clinic (approval number: 20‐002897).

## Results

A total of 445 MOGAD patients with at least 2 years of follow‐up (56.4% female; 49.2% Asian and 47.9% White) were included (Table 1). The median age at MOGAD onset was 28.2 years (interquartile range [IQR], 12.1–48.0), and the median disease duration was 5.4 years (IQR, 3.1–9.1). Sixteen patients (3.6%; 95% CI: 2.2%–5.6%; South Korea, 12; Mayo Clinic, 4) with MOGAD had a concurrent neoplasm, including two previously reported cases,^11^, ^12^ all of whom were adults. The diagnosis of neoplasm was made within 1 year before or after MOGAD onset in 14/16 patients (87.5%), and preceded the onset of MOGAD in 9/16 (56.3%) (Fig. 1B).

**Table 1:** Baseline demographics, neoplasm frequency, and standardized incidence ratio of concurrent neoplasm in MOGAD patients with at least 2 years of follow‐up.

Baseline demographics	Total patients (*n* = 445)	Mayo Clinic (*n* = 230)	South Korea (*n* = 215)	*P*‐value
Female, *n* (%)	251 (56.4)	142 (61.7)	109 (50.7)	0.022
Race, *n* (%)				
Asian	219 (49.2)	5 (2.2)	214 (99.5)	<0.001
Black	8 (1.8)	8 (3.5)	0 (0.0)	0.008
White	213 (47.9)	212 (92.2)	1 (0.5)	<0.001
Others	5 (1.1)	5 (2.2)	0 (0.0)	0.062
Adult‐onset MOGAD, *n* (%)	292 (65.6)	152 (66.1)	140 (65.1)	0.842
Age at onset, years, median (IQR)	28.2 (12.1–48.0)	30.8 (13.1–47.4)	26.0 (11.4–49.1)	0.632
Pediatric‐onset MOGAD (<18 years), *n* (%)	153 (34.4)	78 (33.9)	75 (34.9)	0.842
Adult‐onset MOGAD (≥18 years), *n* (%)	292 (65.6)	152 (66.1)	140 (65.1)	0.842
Older‐adult‐onset MOGAD (≥50 years), *n* (%)	100 (22.5)	49 (21.3)	51 (23.7)	0.571
Age at last follow‐up, years, median (IQR)	35.5 (20.4–53.9)	38.0 (23.5–53.9)	32.8 (18.9–55.1)	0.136
MOGAD disease duration, years, median (IQR)	5.4 (3.1–9.1)	6.0 (3.7–10.2)	4.7 (2.7–8.3)	<0.001
Neoplasm at any point in life, *n* (%)	29 (6.5)	10 (4.3)	19 (8.8)	0.082
Concurrent neoplasm within 2 years of MOGAD onset, *n* (%)	16 (3.6)	4 (1.7)	12 (5.6)	0.040
In pediatric‐onset MOGAD (< 18 years)	0 (0.0)	0 (0.0)	0 (0.0)	>0.999
In adult‐onset MOGAD (≥ 18 years)	16 (5.5)	4 (2.6)	12 (8.6)	0.037
In older‐adult onset MOGAD (≥ 50 years)	12 (12.0)	3 (6.1)	9 (17.6)	0.122
Standardized incidence ratio; (95% CI: *p*‐value)				
All MOGAD	3.10 (1.77–4.81, <0.001)	1.65 (0.43–3.65, 0.227)	4.40 (2.26–7.23, < 0.001)	–
Adult‐onset MOGAD	3.18 (1.82–4.94, <0.001)	1.69 (0.44–3.76, 0.214)	4.51 (2.32–7.43, <0.001)	–

Abbreviations: IQR, interquartile range; MOGAD, myelin oligodendrocyte glycoprotein antibody‐associated disease; *n*, number.

In our combined cohort, the SIR was increased at 3.10 (95% CI: 1.77–4.81; *P* < 0.001). SIR elevation was influenced by the South Korean cohort (SIR, 4.40; 95% CI: 2.26–7.23; *P* < 0.001), while trended to be high without statistical significance in the Mayo Clinic cohort (SIR, 1.65; 95% CI: 0.43–3.65; *P* = 0.227) (Table 1). In the sensitivity analyses, both in the 2‐year pre‐MOGAD (n = 622) and 2‐year post‐MOGAD (*n* = 445) periods, the SIRs were increased at 2.74 (95% CI: 1.24–4.82; *P* = 0.007) and at 2.58 (95% CI: 1.02–4.85; *P* = 0.021), respectively. Among MOGAD patients with 1 year of follow‐up (n = 529), the SIR was also elevated at 4.16 (95% CI: 2.27–6.63; *P* < 0.001) (Table 2).

**Table 2:** Standardized incidence ratio (SIR) of concurrent neoplasm in MOGAD.

Cohorts	Number of patients	Observed number of patients with concurrent neoplasm, *n* (%)	Expected number of patients with neoplasm, *n*	SIR	95% CI^a^	*p‐*value^a^	Neoplasm incidence rate, per 100 py	Difference in neoplasm incidence rate (observed–expected), per 100 py
Total patients								
All MOGAD	445	16 (3.6)	5.16	3.10	1.77–4.81	<0.001	0.90	+0.61
Adult‐onset MOGAD	292	16 (5.5)	5.03	3.18	1.82–4.94	<0.001	1.37	+0.94
Mayo clinic cohort								
All MOGAD	230	4 (1.7)	2.43	1.65	0.43–3.65	0.227	0.43	+0.17
Adult‐onset MOGAD	152	4 (2.6)	2.36	1.69	0.44–3.76	0.214	0.66	+0.27
South Korea cohort								
All MOGAD	215	12 (5.6)	2.73	4.40	2.26–7.23	<0.001	1.40	+1.08
Adult‐onset MOGAD	140	12 (8.6)	2.66	4.51	2.32–7.43	<0.001	2.14	+1.67
Sensitivity analyses								
2 years before MOGAD onset	622	9 (1.4)	3.29	2.74	1.24–4.82	0.007	0.72	+0.46
2 years after MOGAD onset	445	7 (1.6)	2.71	2.58	1.02–4.85	0.021	0.79	+0.48
1 year before and after MOGAD onset	529	14 (2.6)^b^	3.36	4.16	2.27–6.63	<0.001	1.32	+1.01

Abbreviations: MOGAD, myelin oligodendrocyte glycoprotein antibody associated disease; py, person‐year.

^a^ 95% confidence interval (CI) and *p*‐value of the SIR.

^b^ Only includes patients with neoplasm diagnosis within 1 year of MOGAD onset.

The 16 MOGAD patients with concurrent neoplasm had a variety of tumors (Table 3). The median time between neoplasm diagnosis and MOGAD onset was 2.3 months (IQR: 5.7 before to 5.6 after MOGAD onset). The most common presentation of MOGAD was optic neuritis (6/16; 37.5%), and 15/16 (93.8%) had clear positive serum MOG‐IgG, while one (6.3%) had isolated CSF MOG‐IgG‐positivity. Persistent seropositivity was noted in 6/9 (66.7%) with repeated MOG‐IgG testing at a median of 33.5 (IQR: 11.0–70.3) months. None of the tested coexisting neuronal antibodies were positive in all tested patients. Six (37.5%) had relapsing MOGAD and seven (43.8%) received maintenance immunotherapy. After neoplasm treatment, 12 (75.0%) patients were in remission of neoplasm, while 4 (25.0%) had progression of neoplasm (Table S1). Among the 12 patients with neoplasm remission, three (25.0%) experienced a relapse of MOGAD after the remission of the neoplasm; one of these in the setting of immune‐checkpoint inhibitor initiation (case #14, Table 3).^12^ Of the nine neoplasm tissues obtained, none showed MOG immunostaining (Fig. 1C). The PNS‐Care scores of 0–3 confirmed that none of our MOGAD patients with concurrent neoplasm had a paraneoplastic etiology (Table 3).

**Table 3:** Clinical information of the 16 MOGAD patients with concurrent neoplasm.

Case no.	Details of MOGAD									Details of neoplasm			PNS‐Care Score (Clinical/Laboratory/Cancer)^b^
	Age at onset/Sex/Race	Duration of follow‐up (years)	Initial phenotype	MRI findings	CSF findings^a^	Serum MOG‐IgG	CSF MOG‐IgG	Follow‐up Serum MOG‐IgG	No. of attacks/Last maintenance treatment	Type/Stage	Tissue MOG expression	Treatment/Outcome	
1	50s/F/Asian	4.2	Rt ON	CE at Rt optic nerve	WBC 0, Prot 50, OCB neg, MBP neg	MFIr 4.66^c^	Negative	MFIr 2.77^c^	6/MMF	Breast cancer	NA	Tumor resection CCRT/No recurrence neoplasm	0 (0/0/0)
2 (Kwon et al., 2020)	30s/M/White	1.6	Bilateral ON, myelitis	Swelling and CE at bilateral optic nerves, multifocal patchy T2 HSI at T3–T4 and T8‐10 spinal cord	WBC 102, Prot 82, OCB pos, and MBP pos	MFIr 5.15^c^	Not tested	MFIr 2.54^c^	2/None	Primary cutaneous γδ T‐cell lymphoma	Negative	CHOP/No recurrence neoplasm	3 (2/0/1)
3	60s/F/Asian	3.4	Brainstem/cerebellar deficit	T2 HSI and subtle at Rt midbrain	WBC 40, Prot 59, OCB neg, and MBP neg	MFIr 10.12^c^	Not tested	No f/u test	1/None	Chronic myeloid leukemia	NA	Dasatinib, imatinib, and nilotinib/No recurrence neoplasm	2 (2/0/0)
4	60s/F/Asian	4.2	Bilateral ON	CE at bilateral optic nerves, a few T2 HSI in bilateral cerebral WM	WBC 1, Prot 69, OCB neg, and MBP neg	MFIr 4.66^c^	Positive	MFIr 1.39^c^	1/MMF	Colon adenocarcinoma/pT1N1aM0	Negative	Tumor resection Adjuvant XELOX/No recurrence neoplasm	0 (0/0/0)
5	40s/F/Asian	0.3 (expired)	ADEM	T2 HSI in corpus callosum and dentate nucleus	WBC 0, Prot 30.2, OCB	Positive (MFIr NA), no f/u test	Not tested	No f/u test	1/None	Pancreatic cancer	Negative	Tumor resection/Expired	3 (2/0/1)
6	50s/M/Asian	0.5 (expired)	Lt ON	CE at Lt optic nerve	WBC 1, Prot 21.7	Positive (MFIr NA), no f/u test	Not tested	No f/u test	1/None	Intestinal T‐cell lymphoma	Negative	Chemotherapy/Expired	1 (0/0/1)
7	70s/M/Asian	3.6	Brainstem/cerebellar deficit	T2 HSI at Lt pons and bilateral cerebellum	WBC 0, Prot 74.5	MFIr 6.76^d^	Not tested	No f/u test	1/None	Multiple myeloma	NA	VRD + denosumab/No recurrence neoplasm	2 (2/0/0)
8	50s/M/ Asian	3.1	Cerebral cortical encephalitis with seizure	Multifocal T2 HSI at bilateral frontal, Rt temporal lobes and thalamus, swelling of Rt temporo‐parietal cortex, and CE along Rt frontal sulci	WBC 17, Prot 24, and OCB pos	MFIr 5.08^d^	Not tested	No f/u test	1/Rituximab	Renal cell carcinoma	NA	Nephrectomy/No recurrence neoplasm	2 (2/0/0)
9	50s/M/Asian	2.4	Rt ON	CE at Rt optic nerve, a 3.5‐cm partly CE ovoid lesion at Lt precentral gyrus	NA	MFIr 5.56^d^	Not tested	MFIr 3.27^d^	1/None	Rectal cancer/Stage 4	Negative	Neoadjuvant chemotherapy Tumor resection Adjuvant CCRT/Progression of neoplasm	0 (0/0/0)
10	40s/M/Asian	3.9	Brainstem/cerebellar deficit	A few T2 HSI at subcortical WM and brainstem	WBC 33, Prot 38.9, and OCB neg	MFIr 4.66^d^	Not tested	MFIr 1.60^d^	1/None	Small cell lung cancer	Negative	CCRT (etoposide and platinum)/No recurrence neoplasm	3 (2/0/1)
11	30s/F/White	6.9	Brainstem/cerebellar deficit, myelitis, and Lt ON	T2 HSI and faint CE at subcortical and periventricular WM, bilateral MCP, and Lt ventral medulla, CE at Lt optic nerve, T2 HSI and faint CE at C3‐T1 spinal cord	NA	Titer 1:1,000	Not tested	Titer 1:100	4/MMF	Papillary thyroid cancer/Stage 1	NA	Total thyroidectomy Radioiodine therapy/No recurrence neoplasm	2 (2/0/0)
12	50s/F/White	4.6	Rt ON	CE at Rt optic nerve	WBC 2, Prot 69, OCB neg, and MBP pos	Titer 1:100	Not tested	Titer 1:1000	3/MMF	Cystic mesothelioma at abdominal wall*	Negative	Tumor resection/No recurrence neoplasm	0 (0/0/0)
13	60s/F/White	2.9	Rt ON	CE at right optic nerve	WBC 1, Prot 40, and OCB pos	Titer 1:1000	Positive	Titer 1:100	1/None	Pancreatic adenocarcinoma /Stage 1B	NA	Neoadjuvant FOLFIRINOX Neoadjuvant gemcitabine and radiotherapy Pancreaticoduodenectomy with intraoperative radiation/No recurrence neoplasm	0 (0/0/0)
14 (Syc‐Mazurek et al., 2024)	50s/M/White	1.4	Brainstem/cerebellar deficits	T2 HSI at Lt superior colliculus and multiple small lesions at subcortical and periventricular WM with faint CE	WBC 11, Prot 64, and OCB neg	Titer 1:1,000	Positive	Titer 1:1000	7/Tocilizumab	BRAF+ metastatic melanoma/Stage 4	Negative (lymph node tissue with metastasis)	Encorafenib and binimetinib/Lymph node recurrence, bone metastasis Pembrolizumab/No recurrence neoplasm	3 (2/0/1)
15	50s/F/Asian	2.2	Cerebral monofocal deficits	T2 HSI and CE at Lt frontal lobe	WBC 6, Prot 58, and OCB neg	MFIr 1.65	Positive, no f/u test	MFIr 1.17	3/Rituximab	Breast cancer/pT1aN0M0	NA	Neoadjuvant TCHP Tumor resection Trastuzumab and tamoxifen/Progression of neoplasm	3 (2/0/0)
16	60s/F/Asian	0.4	Myelitis	T2 HSI at T7‐T11 spinal cord without CE	WBC 140, Prot 191, and OCB neg	MFIr 3.66^c^	Not tested	No f/u test	1/None	Early gastric cancer type IIb/T1bN2M0	Negative	Radical total gastrectomy Adjuvant TS‐1/No recurrence neoplasm	3 (2/0/1)

Abbreviations: +, positive; ADEM, acute disseminated encephalomyelitis; CCRT, concurrent chemoradiotherapy; CE, contrast enhancement; CHOP, cyclophosphamide, doxorubicin, vincristine, and prednisone; CSF, cerebrospinal fluid; F, female; FOLFIRINOX, folinic acid, fluorouracil, irinotecan, and oxaliplatin; f/u, follow‐up; HSI, high signal intensity; IBI, IgG binding index; Lt, left; M, male; MBP, myelin basic protein; MCP, middle cerebellar peduncle; MMF, mycophenolate mofetil; MOG, myelin oligodendrocyte glycoprotein; MOGAD, myelin oligodendrocyte glycoprotein antibody‐associated disease; NA, not available; OCB, oligoclonal band; ON, optic neuritis; PNS, paraneoplastic neurologic syndrome; Prot, protein; Rt, right; TCHP, docetaxel, carboplatin, trastuzumab, and pertuzumab; VRD, velcade, revlimid, and dexamethasone; WBC, white blood cell; WM, white matter; XELOX, capecitabine plus oxaliplatin.

^a^ White blood cells are displayed as /μL and protein as mg/dL.

^b^ Encephalitis and isolated myelopathy (including myelitis) were categorized as intermediate‐risk phenotypes in the PNS criteria. MOGAD presentations with ADEM, cerebral monofocal or polyfocal deficits, brainstem or cerebellar deficits, and cerebral cortical encephalitis were considered variations of encephalitis, and thus were given a clinical score of 2. Patients with a concurrent tumor other than teratoma and had follow‐up duration <2 years were assigned a cancer score of 1.

^c^ Using FACSCaliber (BD bioscience); positive MOG‐IgG titers were MFI ratio >2.36.

^d^ Using Cytomics FC 500 (Beckman Coulter); positive MOG‐IgG titers were MFI ratio >3.65.

## Discussion

In this large international cohort, concurrent neoplasm was identified in 3.6% of MOGAD patients within 2 years of MOGAD onset, but none demonstrated tumor MOG expression making any paraneoplastic association uncertain. This suggests that universal tumor screening is unnecessary in all new‐onset MOGAD patients. However, age‐appropriate screening and clinically guided investigations for neoplasm in patients with MOGAD should be pursued. Moreover, symptoms suggesting MOGAD should not be ignored in cancer patients. The strengths of this study include the novel comparison to the background cancer rate using SIR, the large cohort of MOGAD patients assessed in two separate world regions, and the relatively large number of tumors tested for MOG expression (Table 4).^20^

**Table 4:** Literature review of MOGAD patients with neoplasm within 2 years of MOGAD onset.

Authors (year)	Neoplasm	Initial MOGAD phenotype	Serum MOG‐IgG titer	CSF MOG‐IgG titer	Follow‐up serum MOG‐IgG titer	Clinical course of MOGAD	Tissue MOG expression
Cherian et al. (2022)^13^	Breast carcinoma	Myelitis	Strong at 1:10 (titration not done)	NA	NA	Monophasic	NA
Cirkel et al. (2021)^14^	Ovarian teratoma	Encephalomyeloradiculitis	1:80 (serum GFAP and ITPR‐1 IgGs negative)	1:2 (concomitant GFAP IgG positive 1:10)	Negative (CSF negative; subsequent CSF ITPR‐1 IgG positive 1:100)	Monophasic	Negative
Cobo‐Calvo et al. (2017)^15^	Ovarian teratoma	ADEM	1:640	NA	NA	Monophasic	NA
Hurtubise et al. (2023)^16^	Thymic hyperplasia	ON	1:80	NA	1:80	Relapsing	NA
Jarius et al. (2016)^17^	Ovarian teratoma	Brainstem/cerebellar deficits, myelitis	1: 10,240	1:64	1:640 (CSF negative)	Monophasic	NA
Li et al. (2020)^18^	Lung adenocarcinoma (EGFR mutation)	Myelitis, ON, brainstem/cerebellar deficits	1:10	1:100	(CSF 1:10, 1:32)	Monophasic	NA
Rodenbeck et al. (2021)^19^	Lung carcinoma (poorly differentiated)	ADEM	1:160	NA	NA	NA	NA
Trentinaglia et al. (2023)^20^	Non‐Hodgkin lymphoma	Brainstem/cerebellar deficits	1:320	NA	NA	Relapsing	NA
Trentinaglia et al. (2023)^20^	Melanoma	Cerebral mono/polyfocal deficits	1:320	NA	1:160	Monophasic	NA
Wildemann et al. (2021)^3^	Ovarian teratoma	ON	1:320	Negative	1:32	Relapsing	Positive
Zhang et al. (2022)^4^	Ovarian teratoma	ADEM	1:10 (after 5 days of IVMP)	NA	NA	Monophasic	Positive

*Note*: Literature review of case reports of MOGAD with neoplasm within 2 years of MOGAD onset from literature through August 2023. The cases from Kwon et al.'s^11^ and Syc‐Mazurek et al.'s^21^ reports were included in our study (Table 3, Cases #2 and #14) and thus are not shown in this table.

Abbreviations: ADEM, acute disseminated encephalomyelitis; CSF, cerebrospinal fluid; GFAP, glial fibrillary acidic protein; ITPR‐1, inositol 1,4,5‐trisphosphate receptor type 1; IVMP, intravenous methylprednisolone; MOG, myelin oligodendrocyte glycoprotein; NA, not available; ON, optic neuritis.

Our results suggest a small association between neoplasm and MOGAD, consistent with its categorization as a lower risk antibody in the PNS criteria.^2^ We found that 14/16 MOGAD patients developed neoplasm within 1 year of MOGAD onset, with even higher SIR than at 2 years, although none demonstrated tumor MOG immunostaining. Notably, two prior MOGAD patients with concurrent teratoma had MOG immunostaining in the tumor suggesting that paraneoplastic MOGAD may occasionally occur.^3^, ^4^ Although there was no teratoma in our study, it is noteworthy that MOG staining was attempted on a variety of tumor tissues, including small cell lung cancer, which is high risk for paraneoplastic syndrome, and all results were negative. We hypothesize that in the setting of cancer, dysregulated self‐immunity against tumor cells might have contributed to MOGAD attacks manifesting concurrently with neoplasm in our study,^22^ rather than direct autoantigen presentation by neoplastic tissues as occurs in PNSs.^23^ Detection bias from increased surveillance in those with MOGAD was another possibility, but the SIR was elevated even in the 2 years before MOGAD diagnosis. Other factors that could have played a role included different methodologies for tumor assessment in our MOGAD cohort than in the reference national databases. There may also be regional differences in frequency of concurrent tumors as South Korea (5.6%) had a higher rate than the USA (1.7%) and a previous Italian cohort (1%).^20^ This may reflect differences between predominantly Asian and White populations or high hospital accessibility in South Korea where 97% of the population have coverage for regular cancer screening with national health insurance.^24^ Our findings differ from paraneoplastic AQP4 + NMOSD in which AQP4 expression in tumor tissue is more frequently reported.^25^

Immunotherapy for MOGAD could potentially influence the incidence of neoplasms and contribute to differences between the groups. However, among the seven cases where neoplasms were identified after the onset of MOGAD, only three patients had received immunotherapy, and the incidence rates were similar between the two cohorts (4/215 [1.9%] in Korea vs. 3/230 [1.3%] at Mayo). Additionally, systemic autoimmune diseases could also affect the incidence of neoplasms, but in our study, none of the MOGAD patients with concurrent neoplasms had coexisting autoimmune diseases. Therefore, the impact of immunotherapy or coexisting autoimmune diseases on our study results is minimal.

Limitations of our study were related to the retrospective design and include potential underreporting of neoplasm and heterogeneity in cancer screening between patients and across different sites. However, our median follow‐up duration of 5.4 years should allow for enough time for cancer to manifest. Moreover, MOG immunostaining was not available in all tumor tissues but only in 9/16 (56%).

In conclusion, concurrent neoplasms were found at a higher frequency than the general population within 2 years of MOGAD onset, but all available tumor tissues were negative for MOG immunostaining suggests that paraneoplastic MOGAD is very rare. Nevertheless, investigation for cancer in MOGAD patients with suggestive features should be considered.

## Author Contributions

YNK, NT, EPF, and S‐MK contributed to the conception and design of the study, and drafting and revising of the manuscript. YNK, NT, YG, SS‐M, JYH, J‐SK, KC, SO, S‐JC, ES, JO, SWK, HYS, BCL, BJK, KSP, J‐JS, SHK, S‐HP, SJP, JJC, EPF, and S‐MK contributed to the acquisition and analysis of data. YNK and NT contributed to the statistical analysis, YG, SS‐M, HYS, BCL, KSP, J‐JS, S‐HP, AZ, CFL, SJP, JJC, EPF, and S‐MK contributed to revise the manuscript for intellectual content. YNK, NT, SHK, S‐HP, AZ, SJP, JJC, EPF, and S‐MK contributed to the interpretation of data; EPF and S‐MK contributed to the study supervision.

## Conflict of interest

YNK received a grant from the National Research Foundation of Korea, Eisai, and Korean Neurological Association; lectured, consulted, and received honoraria from Celltrion, Eisai, GC Pharma, Merck Serono, Roche, Sanofi Genzyme, and CorestemChemon. JJC is a consultant for UCB and Horizon. EPF has served on advisory boards for Alexion, Genentech, Horizon Therapeutics, and UCB. He has received research support from UCB. He received royalties from UpToDate. EPF is a site principal investigator in a randomized clinical trial of Rozanolixizumab for relapsing myelin oligodendrocyte glycoprotein antibody‐associated disease run by UCB. EPF is a site principal investigator and a member of the steering committee for a clinical trial of satralizumab for relapsing myelin oligodendrocyte glycoprotein antibody‐associated disease run by Roche/Genentech. EPF has received funding from the NIH (R01NS113828). EPF is a member of the medical advisory board of the MOG project. EPF is an editorial board member of Neurology, Neuroimmunology and Neuroinflammation, and the Journal of the Neurological Sciences and Neuroimmunology Reports. A patent has been submitted on DACH1‐IgG as a biomarker of paraneoplastic autoimmunity. SMK has lectured, consulted, and received honoraria from Bayer Schering Pharma, Genzyme, Merck Serono, and UCB; received a grant from the National Research Foundation of Korea and the Korea Health Industry Development Institute Research; is an Associate Editor of the Journal of Clinical Neurology. SMK and Seoul National University Hospital has transferred the technology of flow cytometric AQP4‐IgG and MOG‐IgG assay to EONE Laboratory, Korea.

## Supporting information


**Methods S1.** Participating institutions from South Korea.
**Table S1.** Clinical manifestations of MOGAD patients with concurrent neoplasm.

## Data Availability

The data that support the findings of this study are available on request from the corresponding author. The data are not publicly available due to privacy or ethical restrictions.
