# Supplementary material for: Assessment of concurrent neoplasms and a paraneoplastic association in MOGAD
Source: Ann Clin Transl Neurol. 2025 Feb 11;12(4):861–70. doi: 10.1002/acn3.52301 (PMC12040514; doi:10.1002/acn3.52301)
Supplement: Supplementary file 1 — Methods S1. Participating institutions from South Korea. Table S1. Clinical manifestations of MOGAD patients with concurrent neoplasm. [file ACN3-12-861-s001.docx]

**Supplementary Materials**

**eMethods. Participating Institutions from South Korea**

The 9 secondary and tertiary hospitals from South Korea were Seoul National University Hospital, Seoul National University Children's Hospital, Seoul National University Bundang Hospital, Severance Hospital, Samsung Medical Center, Chungnam National University Hospital, Konkuk University Medical Center, Inje University Busan Paik Hospital, and Eunpyeong St. Mary's Hospital.

**eTable 1.** Clinical manifestations of MOGAD patients with concurrent neoplasm

|  | **MOGAD with concurrent cancer (n=16)** |
| --- | --- |
| Age at onset of MOGAD, mean (±SD) | 55.8 (±11.2) |
| Ethnicity, Asian; White; others, n (%) | 11/219 (5.0) ; 5/206 (2.4) ; 0/20 (0.0) |
| Female, n (%) | 9/16 (56.3) |
| Disease duration, median months [range] | 35 [4 - 83] |
| Clinical phenotype |  |
| Optic neuritis, n (%) | 6/16 (37.5) |
| ADEM, n (%) | 1/16 (6.3) |
| Myelitis, n (%) | 1/16 (6.3) |
| Polyfocal or monofocal cerebral deficit, n (%) | 1/16 (6.3) |
| Brainstem / cerebellar deficit, n (%) | 4/16 (25.0) |
| Cortical encephalitis with seizure, n (%) | 1/16 (6.3) |
| Optic neuritis + myelitis/brainstem, n (%) | 2/16 (12.5) |
| Persistent seropositivity, n (%) | 6/9 (66.7) |
| Relapse course, n (%) | 7/16 (43.8) |
| Total number of attack, median [range] | 1 [1 - 7] |

Abbreviations: ADEM, acute disseminated encephalomyelitis; MOGAD, myelin oligodendrocyte glycoprotein antibody associated disease; SD, standard deviation
